# Gram-Negative Bacterial Endotoxin LPS Induces NeuGc Loss through Ets1-Dependent Downregulation of Intestine-Specific *pcmah* Transcript in Porcine Intestinal Cells

**DOI:** 10.3390/ijms21144892

**Published:** 2020-07-10

**Authors:** Choong-Hwan Kwak, Kwon-Ho Song, Cheorl-Ho Kim

**Affiliations:** 1 Molecular and Cellular Glycobiology Unit, Department of Biological Science, Sungkyunkwan University, Seoburo 2066, Jangan-Gu, Suwon, Gyunggi-Do 16419, Korea; kwonho@live.co.kr (K.-H.S.); hahaaaa@nate.com (C.-H.K.); 2 Samsung Advanced Institute for Health Sciences and Technology (SAIHST), Samsung Medical Center, Seoul 06351, Korea

**Keywords:** N-glycolylneuraminic acid (NeuGc), cytidine-5′-monophospho-N-acetylneuraminic acid hydroxylase (CMAH), lipopolysaccharide (LPS), Ets1

## Abstract

N-glycolylneuraminic acid (NeuGc), a non-human sialic acid derivative synthesized by cytidine-5′-monophospho-N-acetylneuraminic acid hydroxylase (CMAH), plays a crucial role in mediating infections by certain pathogens. Although it has been postulated that NeuGc biosynthesis and CMAH expression are downregulated during microbial infection, the underlying mechanisms remain unclear. The present study showed that exposure to lipopolysaccharide (LPS), a Gram-negative bacterial endotoxin, leads to loss of NeuGc biosynthesis in pig small intestinal I2I-2I cells. This LPS-induced NeuGc loss was accompanied by decreased CMAH transcript levels, especially intestine-specific *5′pcmah-1*. Furthermore, LPS suppressed the activity of the Pi promoter responsible for *5′pcmah-1* by inhibiting DNA binding of Est1. These findings provide insight into the regulatory mechanisms of Neu5Gc biosynthesis during pathogenic infectious events, which may represent a host defense mechanism that protects the self against pathogenic bacterial infections even in non-sanitary environments.

## 1. Introduction

Sialic acids are typically found at the non-reducing ends of oligosaccharide chains, which are involved in various biological processes, such as the immune response and infections [1,2]. N-glycolylneuraminic acid (NeuGc) is one of the major types of sialic acid found in most mammals except in humans in physiological state [3]. NeuGc-containing glycoconjugates have been implicated as a crucial mediator in the infection process [4,5,6,7,8]. Indeed, Neu5Gc acts as a target receptor or ligand for microbial pathogens such as *Escherichia coli* K99 and for bacterial toxins such as subtilase cytotoxin secreted by Shiga toxigenic *E. coli* [4,7,8]. Meanwhile, accumulating evidence indicates that the NeuGc level is regulated during the developmental process as well as upon infection by intestinal parasites [4,5,9]. For example, in the pig small intestine, Neu5Gc concentration is maximal at birth and gradually decreases in adults, which may explain the susceptibility of newborn piglets to pig enteric pathogens such as *E. coli* K99 [4,9]. In addition, the NeuGc level is downregulated by the rat intestinal parasite *Nippostrongylus brasiliensis* [5]. These findings raise the obvious question of precisely how NeuGc levels are regulated in the course of infection.

Biosynthesis of NeuGc is mediated by a specific hydroxylase, cytidine-5′-monophospho-N-acetylneuraminic acid hydroxylase (CMAH), which catalytically converts CMP-NeuAc to CMP-NeuGc [10,11,12]. The profile of NeuGc formation has been found to correlate with CMAH abundance in tissues, and CMAH expression is one of the major factors determining NeuGc level [9]. Previous studies have reported that CMAH expression is tissue-dependent and is regulated during infection by certain parasites or bacterial endotoxins [5,9,13]. Although these results indicate that CMAH expression, which is directly related to NeuGc biosynthesis, may be downregulated by certain infectious agents, the underlying mechanisms remain unclear.

Previously, we demonstrated that the pig *CMAH* (*pcmah*) gene has two distinct 5′ alternative splicing forms, *5′pcmah-1* and *5′pcmah-2*, which exhibit intestine-specific and housekeeping expression, respectively [14,15]. In an effort to elucidate the regulatory mechanisms relevant to these *pcmah* transcripts, we identified two distinct promoters, intestine-specific Pi and housekeeping Ph, which are responsible for the expression of *5′pcmah-1* and *5′pcmah-2*, respectively [14,16]. Furthermore, our previous study established that the transcription factor Ets1 is necessary for intestine-specific activity of the Pi promoter [16]. However, it is unclear how *pcmah* is regulated during the infectious process.

The gastrointestinal tract, where many trillions of commensal and infectious bacteria reside, has the highest concentrations of LPS [17,18]. Gut-derived bacterial LPS plays an essential role in inducing intestinal and systemic inflammatory responses, and it has been implicated as a pathogenic factor of necrotizing enterocolitis and inflammatory bowel disease [19]. With regard to NeuGc regulation, it was reported that mRNA expression of CMAH is downregulated by lipopolysaccharide (LPS), which in turn contributes to NeuGc loss, in mouse B cells [13]; however, the underlying mechanisms remain to be elucidated. In this study, we focused on the regulatory mechanisms by which infectious conditions imposed by bacterial endotoxin LPS induce NeuGc loss in pig intestinal epithelial cells. We discovered that LPS-induced NeuGc loss arises through intestine-specific transcriptional regulation of the *pcmah* gene. Therefore, these findings provide insight into a host defense mechanism that protects the self against pathogenic bacterial infections.

## 2. Results

### 2.1. LPS Exposure Leads to Loss of NeuGc Biosynthesis in Pig Small Intestinal IPI-2I Cells

**Figure 1:**
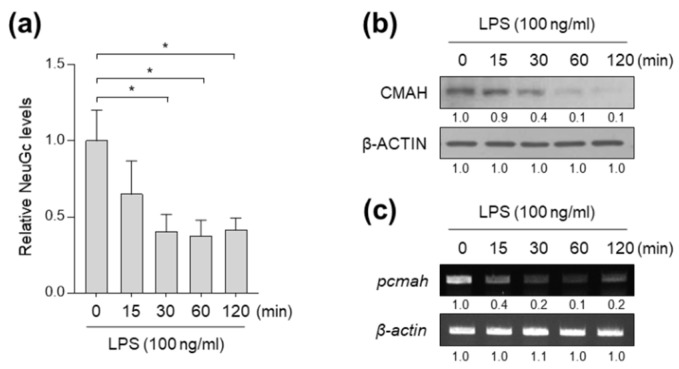
LPS exposure induces NeuGc loss and downregulation of *pcmah*. IPI-2I cells were treated with 100 ng/mL LPS for the indicated times. (**a**) Neu5Gc levels in the cells were determined by ELISA. Graphs represent three independent experiments performed in triplicate. Data represent the mean ± SD. * *P* < 0.05 by two-tailed Student’s t test. (**b**) Protein level of pcmah was analyzed by immunoblotting. β-ACTIN was included as an internal loading control. Numbers below blot images indicate fold-change in protein expression. (**c**) mRNA expression levels of *pcmah* were determined by RT-PCR. Numbers below images indicate fold-change in protein or mRNA level.

To investigate the effects of bacterial endotoxin on NeuGc biosynthesis, pig small intestinal IPI-2I cells were treated with 100 ng/mL LPS. We observed a significant reduction in NeuGc biosynthesis in IPI-2I cells after LPS exposure (Figure 1a). As CMAH expression is a key rate-limiting step in NeuGc regulation, we assessed the levels of CMAH protein and mRNA following LPS treatment. Indeed, LPS treatment resulted in a time-dependent gradual loss of CMAH protein, which was accompanied by decreased levels of *CMAH* mRNA (Figure 1b,c). These results indicate that LPS induces NeuGc loss through a reduction in transcription of the *CMAH* gene in pig small intestinal IPI-2I cells.

### 2.2. LPS-Induced pcmah Loss Is Mediated by the Intestine-Specific 5′pcmah-1 Transcript

**Figure 2:**
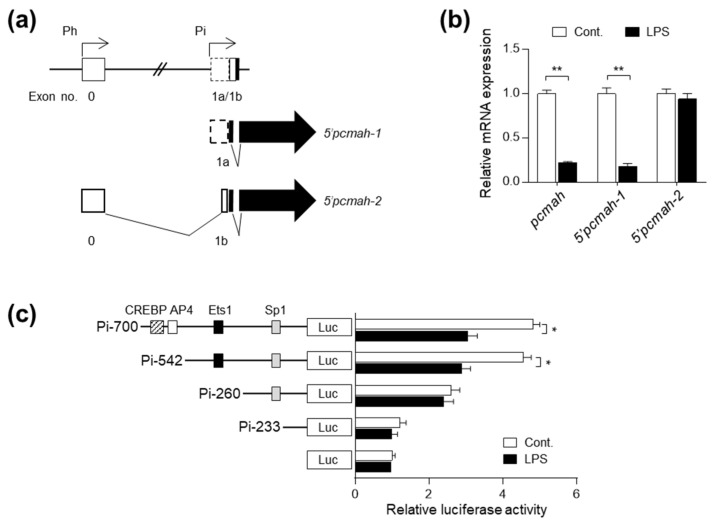
LPS downregulates levels of intestine-specific *5’pcmah-1* transcript and Pi promoter of *pcmah* in IPI-2I cells. (**a**) A schematic diagram for genomic structure of the *pcmah*. *5’pcmah-1* and *5’pcmah-2*, two alternative splicing variants of the *pcmah*, have a distinct transcription initiation site located in exons 0 and 1a, respectively. Each promoter region responsible for intestine specific splicing variant *5’pcmah-1* and housekeeping splicing variant *5’pcmah-2* is indicated by Pi (intestine specific promoter) and Ph (housekeeping promoter), respectively. Shaded boxes indicate the coding exons, while open boxes indicate untranslated exons. Two splicing variants of the *pcmah* share a common ORF region (shaded arrow boxes). (**b**) IPI-2I cells were treated with 100 ng/mL LPS for 1 h. mRNA expression of *pcmah*, *5’pcmah-1*, and *5’pcmah-2* were determined by qRT-PCR. Numbers below images indicate fold-change in mRNA expression. (**c**) The indicated 5’ deletion constructs for the Pi promoter region were transiently transfected into IPI-2I cells. After 24 h, the cells were treated with LPS for 1 h. Luciferase and β-galactosidase activity in the transfected cells was measured. For each transfection, luciferase activity was normalized with β-galactosidase activity and the relative value was determined from the ratio of normalized activity and activity in cells transfected with the empty pGL3-basic vector. Transcription factors in the region are indicated. Graphs represent three independent experiments performed in triplicate. Data represent the mean ± SD. * *P* < 0.05, ** *P* < 0.01 by two-way ANOVA.

Previously, we demonstrated that the pig *cmah* gene has two distinct 5′ alternative splicing forms, *5′pcmah-1* and *5′pcmah-2*, which exhibit intestine-specific and housekeeping expression, respectively [14] (Figure 2a). Given our previous finding that *5′pcmah-1* and *5′pcmah-2* encode an identical pCMAH protein [15] (Figure 2a), we questioned which transcript is responsible for the loss of CMAH protein following LPS treatment in IPI-2I cells. Intriguingly, LPS treatment drastically reduced *5′pcmah-1* but not *5′pcmah-2* transcript levels in IPI-2I cells (Figure 2b), suggesting that LPS-induced pCMAH loss is mediated by a reduction of the intestine-specific transcript rather than the housekeeping transcript. We next attempted to elucidate the underlying mechanism responsible for transcriptional loss of *5′pcmah-1* following LPS exposure. Since *5′pcmah-1* is mainly controlled by the Pi (intestine-specific) promoter [14,16], we examined the effects of LPS treatment on Pi promoter activity using serially constructed 5′-deletion mutants, Pi-700, Pi-542, Pi-260 and Pi-233. The activity of Pi-542 and Pi-700 was markedly decreased upon LPS treatment in IPI-2I cells, while there was no alteration in the activity of Pi-260 or Pi-230 after LPS treatment (Figure 2c). This result indicates that the region between 542 and 260 bp may be responsible for LPS-induced transcriptional loss of *5′pcmah-1*.

### 2.3. LPS Interferes with Ets1 Binding to the Pi Promoter Region of pcmah

**Figure 3:**
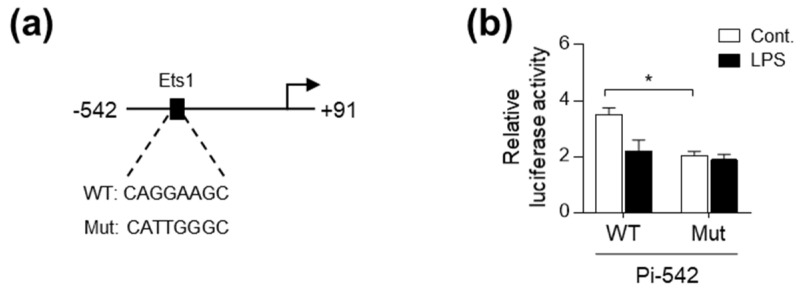
The Ets1 binding element is crucial for LPS-induced repression of Pi activity. (**a**) Nucleotide sequences of the Ets1 binding site (WT) and mutated bases (Mut) in Pi-542 are shown. (**b**) Luciferase activity of WT and Mut Pi-542 in IPI-2I cells with or without LPS treatment (100 ng/mL, 1 h). Graphs represent three independent experiments performed in triplicate. Data represent the mean ± SD. * *P* < 0.05 by one-way ANOVA.

In an effort to identify the LPS-responsive element in the Pi promoter, we noted that genetic deletion of the region containing the Ets1 binding site, from Pi-542 to Pi-260, resulted in a failure to decrease Pi promoter activity after LPS treatment (Figure 2b). In this regard, we previously demonstrated that the Ets1 binding element is essential for the basal activity of the Pi promoter, which in turn contributes to intestine specific expression of *5’pcmah-1* in IPI-2I cells [16]. Given the crucial role of Ets1 binding element in an intestine-specific activity of the Pi promoter [16], we questioned that the Ets1 binding element may also act as an LPS-responsive element in the Pi promoter. To investigate the role of Ets1 binding element in the suppressive effect of LPS on Pi promoter activity, we engineered a mutant form of the Pi-542 construct (Pi-542-Mut) in which the Ets1-binding element has been mutated (Figure 3a). Consistent with our previous results [16], mutation of the Ets1 binding site on Pi-542 significantly decreased the activity of Pi-542 in intestinal IPI-2I cells (Figure 3a,b). However, LPS treatment did not further reduce the activity of the Pi-542-Mut construct (Figure 3b).

**Figure 4:**
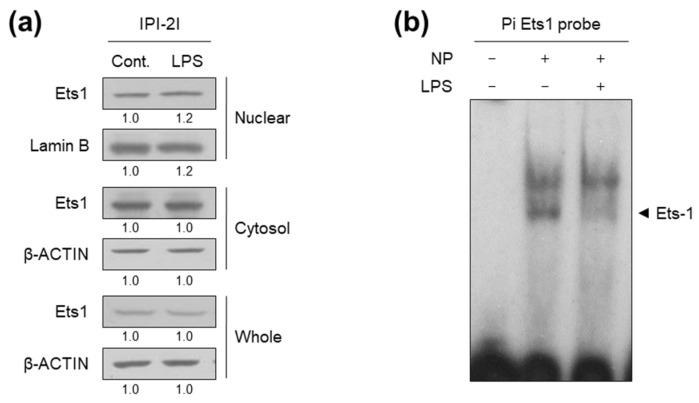
LPS interferes with Ets1 binding to the Pi promoter region of *pcmah*. IPI-2I cells were treated with 100 ng/mL LPS for 1 h. (**a**) Nuclear and cytosol fractions and whole cell lysates were analyzed by immunoblot analysis with the indicated antibodies. Numbers below images indicate fold-change in expression levels. (**b**) Nuclear extracts of IPI-2I cells treated with or without LPS were incubated with a ^32^P-labeled oligonucleotide probe (Pi Ets1). The reaction mixture was then examined by EMSA as described in the Materials and Methods Section. “NP” refers to nuclear protein. Arrows indicate the specific binding complex for Ets1.

These results indicate that the Ets1 binding element on the Pi promoter is important for the activity in response to LPS as well as basal promoter activity. Previously, we showed that Ets-1 transcription factor is abundant in intestinal tissues, which in turn activates the Pi promoter, thereby contributing to intestine-specific expression of *pcmah* [16]. However, there was no alteration in Ets1 protein abundance in either the nuclear or whole protein fractions of LPS-treated IPI2I cells compared with control (Figure 4a). It has been documented that intracellular signaling can inhibit the DNA-binding activity of Ets1 [20]. To determine whether LPS affects binding of Ets1 to its binding element in the Pi promoter, an electrophoretic mobility shift assay (EMSA) was performed using nuclear extracts of IPI-2I cells treated with or without LPS, and a ^32^P-labeled oligonucleotide probe containing Ets1 binding element of the Pi promoter region (Pi Ets1 probe). In this regard, a specific binding of Ets1 protein to the Pi Ets1 probe has been previously demonstrated [16]. Notably, nuclear protein (NP) isolated from IPI-2I cells successfully bound to the Pi-Ets1 probe; however, NP of LPS-treated IPI-2I cells failed to complex with the oligonucleotide probe (Figure 4b). The result indicates a suppressive role of LPS on the Ets1-oligonucleotide complex in IPI-2I cells. Taken together, our findings suggest that LPS may inhibit DNA binding of Ets1, thereby suppressing the activity of the Pi promoter responsible for *5’pcmah-1* in IPI-2I cells.

## 3. Discussion

Intestinal epithelial cells, which line the gastrointestinal tract, function as a barrier against foreign parasitic infections. NeuGc-containing glycoconjugates on the surface of intestinal epithelial cells have been implicated as the primary compounds that interact with microbes or bacterial endotoxins during the infection process [4,5,6,7,8,9]. Although NeuGc loss was previously observed in the microbial infection process [4,5], the underlying mechanisms remain unclear. Here, we show that NeuGc loss can be triggered by the bacterial endotoxin LPS via transcriptional repression of the *pcmah* gene, in particular its intestine-specific transcript (*5’pcmah-1*).

LPS concentrations are highest in the gut lumen, where many trillions of commensal bacteria reside [17,18]. In general, however, LPS in the gut lumen do not penetrate across the healthy intestinal epithelium [21]. For instance, while the concentration of LPS in the gut lumen has been reported to be 1.8 μg/mL in the rat distal ileum [22], previous reports suggested plasma LPS levels of 0.1–1 ng/mL to be physiologically relevant [23,24]. In this regard, we found that at least 50 ng/mL of LPS is required to effectively inhibit the levels of CMAH protein and mRNA at our experimental conditions (data not shown). Therefore, the evidence that high dose (100 ng/mL) of LPS effectively inhibits NeuGc biosynthesis may be relevant to the pathological context rather than physiological conditions.

Given the crucial role of NeuGc as a target receptor for certain pathogens and toxins in the infection process, it is intriguing, and perhaps counterintuitive, that LPS exposure leads to loss of NeuGc biosynthesis. Nonetheless, it has been reported that the level of NeuGc-containing glycoconjugates is maximal at birth and gradually decreases in the later stages of an infection [4]. This may explain the observation that newborn pigs are particularly susceptible to pig enteric pathogens [4]. In light of this, we postulate that NeuGc loss in intestinal epithelial cells following LPS exposure may be a defense mechanism against infection.

The transcription factor Ets1 is widely expressed in developing and mature intestines and is closely related to certain inflammatory disease processes [25]. We recently demonstrated that Ets1 plays an important role in the activity of the Pi promoter responsible for intestine-specific expression of *pcmah* [16]. While we showed that relatively high levels of Ets1 confer intestine-specificity of *pcmah* expression [16], LPS treatment did not affect Ets1 protein levels in nuclear or whole cell lysates of IPI-2I cells (Figure 4a). Nonetheless, it has been documented that intracellular signaling can inhibit the DNA-binding activity of Ets1 [20,26,27]. Indeed, the DNA-binding activity of Ets-1 was significantly reduced in LPS-stimulated human macrophage THP-1 cells [28]. Calcium-induced phosphorylation of Ets-1 also causes a reduction in its DNA-binding activity [26]. These results raise the obvious question of precisely how LPS could interfere Ets1 function. In this regard, it is well known that binding of the LPS to specific cellular receptors, including toll-like receptor 4 (TLR4), triggers a downstream signaling cascade leading to transcriptional regulation of numerous genes [29]. In line with these results, our findings suggest that LPS signaling may inhibit DNA binding of Ets1, thereby suppressing the activity of the Pi promoter responsible for *5’pcmah-1*. Nevertheless, it will be important in future studies to assess the precise underlying mechanisms by which LPS signaling interferes Ets1 function.

Although our data clearly show that LPS treatment decreases mRNA and protein expression of pig CMAH, it is important to note that, while NeuGc levels were reduced by LPS exposure, considerable amounts of NeuGc remained (Figure 1a). One possible explanation for the remaining NeuGc involves uptake and utilization of exogenous NeuGc. Indeed, it is well known that fetal bovine serum is a good exogenous source of NeuGc. Nonetheless, we cannot exclude possible contributions of housekeeping splicing variant *5’pcmah-2* as a compensatory mechanism and other regulatory mechanisms responsible for NeuGc biosynthesis.

Altogether, our data provide evidence that infectious conditions, such as that imposed by the bacterial endotoxin LPS, lead to a loss of NeuGc synthesis in intestinal epithelial cells. When porcine intestinal cells meet the pathogenic bacterial endotoxin LPS, the cells begin to respond by recognizing the bacterial infection and decreasing the concentration of NeuGc on the cell surface via downstream signaling. In the process, reduction in NeuGc levels is brought about through intestine-specific transcriptional regulation of the *pcmah* gene, in particular *5’pcmah-1*. In turn, this loss of NeuGc in pig intestinal epithelial cells may alter the binding activities of any pathogen that employs NeuGc for any part of its pathogenic process. Therefore, these findings provide insight into the regulatory mechanisms of Neu5Gc biosynthesis during pathogenic infectious events, which may represent a host defense mechanism that protects the self against pathogenic bacterial infections even in non-sanitary environments.

## 4. Materials and Methods

### 4.1. Cell Culture

The IPI-2I cell line, derived from pig small intestinal tissue, was obtained from the European Collection of Cell Culture (ECACC, Salisbury, UK) and cultured in DMEM (WelGENE, Daegu, Korea) containing insulin (0.024 IU/mL, Sigma Aldrich, St Louis, MO, USA), glutamine (4 mM, Sigma Aldrich) and 100 unit/mL penicillin–streptomycin (WelGENE). The cells were grown at 37 °C in a 5% CO_2_ incubator/humidified chamber.

### 4.2. Enzyme-Linked Immunosorbent Assay (ELISA) for NeuGc

Neu5Gc levels were measured with an ELISA assay kit using chicken polyclonal IgY (BioLegend, Sandiego, CA, USA), as described previously [14].

### 4.3. Western Blot Analysis

Lysates extracted from a total of 2.5 × 10^5^ cells were used to perform Western blot. Primary antibodies against CMAH, ETS1 and Lamin B (Santa Cruz Biotechnology, CA, USA) as well as β-ACTIN (Sigma Aldrich) were used. Western blotting was followed by the appropriate secondary antibodies conjugated with horseradish peroxidase. Immunoreactive bands were developed with the chemiluminescence ECL detection system (Sigma-Aldrich) and signals were detected using ChemiDoc (Davinchi-K, Seoul, Korea).

### 4.4. Reverse Transcription-Polymerase Chain Reaction and Real-Time Quantitative PCR

Total RNA was isolated using TRIZOL reagent (Invitrogen, Carlsbad, CA, USA), and cDNA was synthesized using AccuPower® RT-PreMix (Bioneer, Daejon, Korea), according to the manufacturer’s recommended protocol. PCR was performed with the following specific primers: pcmah, 5′-ATGAGCAGCATCGAACAAAC-3′ (forward) and 5′-ACAACCAGTTCGTCTTGACA-3′ (reverse); 5′pcmah-1, 5′-GTCAACGGAAATACTGAGCTGGGT-3′ (forward) and 5′-TCGTCTTGACAGAAGCTTCCAGGA-3′ (reverse); 5′pcmah-2, 5′-TGCTTCTCCAGGGGCGAAACC-3′ (forward) and 5′-TCGTCTTGACAGAAGCTTCCAGGA-3′ (reverse); β-actin, 5′-CACGCCATCCTGCGTCTGGA-3′ (forward) and 5′-TCTGCATCCTGTCGGCGATG-3′ (reverse). For generalization of the obtained data, equal amounts of mRNA were used. Expression levels of β-actin as an internal control were analyzed and confirmed. Real-time quantitative RT-PCR was performed using iQ SYBR Green super mix (Bio-Rad) with the specific primers on a CFX96 real-time PCR detection system. All real-time quantitative PCR experiments were performed in triplicate and quantification cycle (Cq) values were determined using Bio-Rad CFX96 Manager 3.0 software. Relative quantifications of the mRNA levels were performed using the comparative Ct method with β-ACTIN as the reference gene.

### 4.5. DNA Constructs and Site-Directed Mutagenesis

The DNA constructs corresponding to the intestine-specific (Pi) promoter of the pig CMAH gene have been described previously [14,16]. Site-directed mutagenesis was performed using a QuickChange XL Site-directed Mutagenesis kit (Stratagene, San Diego, CA, USA) according to the manufacturer’s instructions. To create mutations in the Ets1-binding sites of the Pi promoter region, the following primer set was used: 5′-TATGCCACATTGGGCAGCCCT-3′ (forward) and 5′-AGGGCTGCCCAATGTGGCATA-3′ (reverse). The mutant construct was confirmed by DNA sequencing.

### 4.6. Luciferase Reporter Assay

For the luciferase assay, IPI-2I cells were co-transfected with 0.25 pmol of the indicated Ph or Pi promoter constructs and 0.25 µg of β-galactosidase reporter plasmid using polyethylenimine transfection reagent (Polyscienses, Warrington, PA, USA). After 24 h, the cells were treated with LPS (100 ng/mL, Sigma) for 1 h. Luciferase activity and β-galactosidase activity were determined using a luciferase reporter assay system kit (Promega, Madison, WI, USA). Luciferase activity was normalized to β-galactosidase activity.

### 4.7. Electromobility Shift Assay (EMSA)

For EMSA, a single-stranded oligonucleotide was commercially synthesized using IDT DNA (IDT, IA, USA), as follows: Pi Ets-1, 5′-TATGCCACAGGAAGCAGCCCT-3′. To anneal the double strand probes, complementary oligonucleotides were added together in a 1:1 molar ratio and incubated at 95 °C for 2 min and at 25 °C for 45 min. The EMSA experiment was performed using a gel shift assay system kit (Promega, USA), as previously described [14].

### 4.8. Statistical Analysis

All data are representative of at least three separate experiments. Statistical differences were calculated by Student’s t-test (two-tailed, unpaired), one-way ANOVA or two-way ANOVA using GraphPad Prism software. A P-value of less than 0.05 was considered statistically significant.

## 5. Conclusions

**Figure 5:**
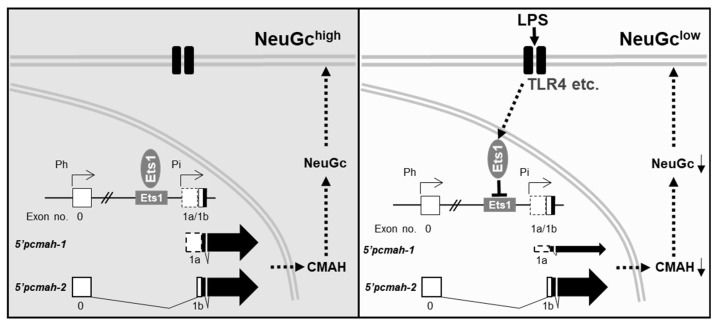
Schematic interpretation of the molecular mechanism by which LPS induces NeuGc loss in pig intestinal tissues.

The present study demonstrated to the regulatory mechanisms by which LPS induces NeuGc loss in pig small intestinal I2I-2I cells (Figure 5). The LPS-induced NeuGc loss was accompanied by decreased CMAH transcript levels, especially intestine-specific 5’pcmah-1. In this process, the transcription factor Ets1 played a crucial role in the downregulation of 5’pcmah-1 following LPS treatment. We postulate that LPS may interfere the Ets1 function via intracellular signaling pathway by binding to specific cellular receptors, including toll-like receptor 4 (TLR4). Indeed, it was shown that LPS interferes with Ets1 binding to DNA, thereby suppressing the activity of the Pi promoter responsible for 5’pcmah-1. Therefore, these results provide insight into the regulatory mechanisms of Neu5Gc biosynthesis during pathogenic infectious events.

## Abbreviations

NeuGc	N-glycolylneuraminic acid
CMAH	Cytidine-5′-monophospho-N-acetylneuraminic acid hydroxylase
LPS	Lipopolysaccharide
ELISA	Enzyme-linked immunosorbent assay
RT-PCR	Reverse transcription-polymerase chain reaction
EMSA	Electromobility shift assay
Pi	Intestine specific promoter

## References

[1] Angata T., Varki A. (2002). Chemical diversity in the sialic acids and related α-keto acids: An evolutionary perspective. Chem. Rev..

[2] Kelm S., Schauer R. (1997). Sialic acids in molecular and cellular interactions. International Review of Cytology.

[3] Chou H.-H., Takematsu H., Diaz S., Iber J., Nickerson E., Wright K.L., Muchmore E.A., Nelson D.L., Warren S.T., Varki A. (1998). A mutation in human CMP-sialic acid hydroxylase occurred after the Homo-Pan divergence. Proc. Natl. Acad. Sci. USA.

[4] Teneberg S., Willemsen P., de Graaf F.K., Karlsson K.-A. (1990). Receptor-active glycolipids of epithelial cells of the small intestine of young and adult pigs in relation to susceptibility to infection with Escherichia coli K99. FEBS Lett..

[5] Karlsson N.G., Olson F.J., Jovall P.-Å., Andersch Y., Enerbäck L., Hansson G.C. (2000). Identification of transient glycosylation alterations of sialylated mucin oligosaccharides during infection by the rat intestinal parasite Nippostrongylus brasiliensis. Biochem. J..

[6] Rolsma M.D., Kuhlenschmidt T.B., Gelberg H.B., Kuhlenschmidt M.S. (1998). Structure and function of a ganglioside receptor for porcine rotavirus. J. Virol..

[7] Byres E., Paton A.W., Paton J.C., Löfling J.C., Smith D.F., Wilce M.C., Talbot U.M., Chong D.C., Yu H., Huang S. (2008). Incorporation of a non-human glycan mediates human susceptibility to a bacterial toxin. Nature.

[8] Willemsen P., De Graaf F. (1993). Multivalent binding of K99 fimbriae to the N-glycolyl-GM3 ganglioside receptor. Infect. Immun..

[9] Malykh Y.N., King T.P., Logan E., Kelly D., Schauer R., Shaw L. (2003). Regulation of N-glycolylneuraminic acid biosynthesis in developing pig small intestine. Biochem. J..

[10] Kawano T., Koyama S., Takematsu H., Kozutsumi Y., Kawasaki H., Kawashima S., Kawasaki T., Suzuki A. (1995). Molecular cloning of cytidine monophospho-N-acetylneuraminic acid hydroxylase. Regulation of species-and tissue-specific expression of N-glycolylneuraminic acid. J. Biol. Chem..

[11] Muchmore E., Milewski M., Varki A., Diaz S. (1989). Biosynthesis of N-glycolyneuraminic acid. The primary site of hydroxylation of N-acetylneuraminic acid is the cytosolic sugar nucleotide pool J. Biol. Chem..

[12] Schlenzka W., Shaw L., Kelm S., Schmidt C.L., Bill E., Trautwein A.X., Lottspeich F., Schauer R. (1996). CMP-N-acetylneuraminic acid hydroxylase: The first cytosolic Rieske iron-sulphur protein to be described in Eukarya. FEBS Lett..

[13] Naito Y., Takematsu H., Koyama S., Miyake S., Yamamoto H., Fujinawa R., Sugai M., Okuno Y., Tsujimoto G., Yamaji T. (2007). Germinal center marker GL7 probes activation-dependent repression of N-glycolylneuraminic acid, a sialic acid species involved in the negative modulation of B-cell activation. Mol. Cell. Biol..

[14] Song K.-H., Kwak C.-H., Jin U.-H., Ha S.-H., Park J.-Y., Abekura F., Chang Y.-C., Cho S.-H., Lee K., Chung T.-W. (2016). Housekeeping promoter 5′pcmah-2 of pig CMP-N-acetylneuraminic acid hydroxylase gene for NeuGc expression. Glycoconj. J..

[15] Song K.-H., Kang Y.-J., Jin U.-H., Park Y.-I., Kim S.-M., Seong H.-H., Hwang S., Yang B.-S., Im G.-S., Min K.-S. (2010). Cloning and functional characterization of pig CMP-N-acetylneuraminic acid hydroxylase for the synthesis of N-glycolylneuraminic acid as the xenoantigenic determinant in pig–human xenotransplantation. Biochem. J..

[16] Song K.-H., Kwak C.-H., Chung T.-W., Ha S.-H., Park J.-Y., Ha K.-T., Cho S.-H., Lee Y.-C., Kim C.-H. (2019). Intestine specific regulation of pig cytidine-5′-monophospho-N-acetylneuraminic acid hydroxylase gene for N-glycolylneuraminic acid biosynthesis. Sci. Rep..

[17] Metzler-Zebeli B.U., Mann E., Schmitz-Esser S., Wagner M., Ritzmann M., Zebeli Q. (2013). Changing dietary calcium-phosphorus level and cereal source selectively alters abundance of bacteria and metabolites in the upper gastrointestinal tracts of weaned pigs. Appl. Environ. Microbiol..

[18] Seki E., Schnabl B. (2012). Role of innate immunity and the microbiota in liver fibrosis: Crosstalk between the liver and gut. J. Physiol..

[19] Guo S., Nighot M., Al-Sadi R., Alhmoud T., Nighot P., Ma T.Y. (2015). Lipopolysaccharide regulation of intestinal tight junction permeability is mediated by TLR4 signal transduction pathway activation of FAK and MyD88. J. Immunol..

[20] Dittmer J. (2003). The biology of the Ets1 proto-oncogene. Mol. Cancer.

[21] Benoit R., Rowe S., Watkins S.C., Boyle P., Garrett M., Alber S., Wiener J., Rowe M.I., Ford H.R. (1998). Pure endotoxin does not pass across the intestinal epithelium in vitro. Shock.

[22] Yagi S., Takaki A., Hori T., Sugimachi K. (2002). Enteric lipopolysaccharide raises plasma IL-6 levels in the hepatoportal vein during non-inflammatory stress in the rat. Fukuoka Igaku Zasshi.

[23] Guo S., Al-Sadi R., Said H.M., Ma T.Y. (2013). Lipopolysaccharide causes an increase in intestinal tight junction permeability in vitro and in vivo by inducing enterocyte membrane expression and localization of TLR-4 and CD14. Am. J. Pathol..

[24] Hurley J.C. (1995). Endotoxemia: Methods of detection and clinical correlates. Clin. Microbiol. Rev..

[25] Oliver J.R., Kushwah R., Hu J. (2012). Multiple roles of the epithelium-specific ETS transcription factor, ESE-1, in development and disease. Lab. Invest..

[26] Rabault B., Ghysdael J. (1994). Calcium-induced phosphorylation of ETS1 inhibits its specific DNA binding activity. J. Biol. Chem..

[27] Cowley D.O., Graves B.J. (2000). Phosphorylation represses Ets-1 DNA binding by reinforcing autoinhibition. Genes Dev..

[28] Suriano A.R., Sanford A.N., Kim N., Oh M., Kennedy S., Henderson M.J., Dietzmann K., Sullivan K.E. (2005). GCF2/LRRFIP1 represses tumor necrosis factor alpha expression. Mol. Cell. Biol..

[29] West A.P., Koblansky A.A., Ghosh S. (2006). Recognition and signaling by toll-like receptors. Annu. Rev. Cell Dev. Biol..

